# Efficacy of anti–tumor necrosis factor therapy for extra-articular manifestations in patients with ankylosing spondylitis: a meta–analysis

**DOI:** 10.1186/s12891-015-0489-2

**Published:** 2015-02-10

**Authors:** Dan Wu, Ying-Ying Guo, Nan-Nan Xu, Shuai Zhao, Lin-Xin Hou, Ting Jiao, Ning Zhang

**Affiliations:** Second Departments of Rheumatology, Shengjing Hospital of China Medical University, 39, Huaxiang Road, Tiexi District, Shenyang, 110022, Liaoning China; First Departments of Rheumatology, Shengjing Hospital of China Medical University, 39, Huaxiang Road, Tiexi District, Shenyang, 110022 Liaoning China

**Keywords:** Ankylosing spondylitis, Anti-TNF therapy, Extra–articular manifestations, Uveitis, Inflammatory bowel disease, Meta–analysis

## Abstract

**Background:**

We performed a meta-analysis to evaluate the effect of anti–tumor necrosis factor (TNF) therapy on the frequency of extra–articular manifestations (EAMs) in patients with ankylosing spondylitis (AS).

**Methods:**

We searched with the terms ‘ankylosing spondylitis’, ‘infliximab’, ‘etanercept’, ‘adalimumab’, ‘golimumab’, ‘certolizumab’, ‘TNF inhibitor/blocker/antagonists’ or ‘anti-TNF’ on MEDLINE, EMBASE and Cochrane Library for randomized controlled trials (RCTs) of ≥12 weeks with parallel or crossover design of TNF inhibitor versus placebo to treat uveitis, inflammatory bowel disease (IBD) and/or psoriasis of AS, published before February 2014.

**Results:**

We found 8 RCTs that fit our criteria. Anti–TNF therapy was associated with less uveitis than placebo in patients with AS (OR: 0.35, 95% CI: 0.15–0.81, *P* = 0.01). Subgroup analysis showed receptor fusion proteins were more efficacious for uveitis than placebo (OR: 0.30, 95% CI: 0.09–0.94, *P* = 0.04), but monoclonal antibodies were not (OR: 0.43, 95% CI: 0.12–1.49, *P* = 0.18). Anti–TNF therapy and placebo group did not significantly differ in treating IBD in AS patients (OR: 0.75, 95% CI: 0.25–2.29, *P* = 0.61). In subgroup analysis, neither monoclonal antibodies (OR: 0.45, 95% CI: 0.10–1.92, *P* = 0.28) nor receptor fusion proteins (OR: 1.52, 95% CI: 0.25–9.25, *P* = 0.65) significantly differed from placebo in treating IBD. We found no suitable reports on psoriasis.

**Conclusions:**

Anti–TNF therapy was preventive for flares or new onset of uveitis in AS patients, and might be an alternative for these patients. However, monoclonal anti–TNF antibodies and TNF receptor fusion proteins were not efficacious for IBD in AS patients.

## Background

Ankylosing spondylitis (AS) is a chronic, progressive, inflammatory rheumatic disease that primarily affects the sacroiliac joints [1]. AS is primarily a disease of the axial skeleton, but some patients have peripheral joint involvement [2]. AS also has some extra–articular manifestations (EAMs). An epidemiological study in Belgium found that 42% of patients with definite AS had one or more EAMs [3]. EAMs mostly occurred in the eye (uveitis), gastrointestinal tract (inflammatory bowel disease, IBD) and skin (psoriasis) [4].

Uveitis, which is characterized by pain with red eye and photophobia, increased tear production and blurring of vision [5], occurs in approximately 20–30% of AS patients during the course of their disease, and is considered the most common EAM [6,7]. Non–steroidal anti–inflammatory drugs (NSAIDs) can only relieve uveitis symptoms for a short period in AS patients, but cannot change the course of their disease or prevent structural damage. NSAIDs treatment can also increase the tendency towards osteoporosis if used for a longer period of time. Some evidence indicates that disease–modifying anti–rheumatic drugs (DMARDs) can reduce uveitis recurrence [8,9]. TNF is present at high concentrations in both aqueous humor and serum of patients with uveitis [10], and may participate actively in the pathogenesis of uveitis. In recent years, several trials have demonstrated the efficacy of anti–TNF therapy in reducing acute uveitis [11,12].

IBD is characterized by a chronic inflammation of the gut mucosa and includes Crohn’s disease (CD) and ulcerative colitis (UC). A recent systematic review and meta-analysis showed the pooled prevalence of IBD in patients with AS to be 6.8% [7]. Traditionally, treatment of IBD has relied on corticosteroids to reduce flares and on immunomodulators to maintain remission [13]. Aminosalicylic acid is widely used to treat UC, but its use in CD is controversial [14]. Several recent trials have demonstrated the efficacy of anti-TNF therapy in reducing IBD [15].

Psoriasis is a systemic inflammatory cutaneous disease with plaque lesions and nail deformities. The pooled prevalence of psoriasis, a secondary disorder in AS, was 9.3% in patients with AS [7].

In a 2010 update by the Assessments in Ankylosing Spondylitis International Society and the European League against Rheumatism (ASAS/EULAR) of recommendations for the management of AS [16], NSAIDs are considered the first-line drug treatment for AS patients with pain and stiffness; DMARDs and intra–articular injections of glucocorticoids in patients with peripheral arthritis may also be considered, although there is no evidence to support the use of these medications for axial diseases; anti-TNF therapy is another option for patients with persistently high disease activity despite conventional treatments.

Infliximab (INF) is a chimeric mouse–human monoclonal immunoglobulin G (IgG) 1 antibody [17]. Adalimumab (ADA) [18], golimumab (GOL) [18] and certolizumab (CZP) [19] are humanized monoclonal anti-TNF-α antibodies. Etanercept (ETA) [20] is a dimeric fusion protein of the TNF receptor linked to the Fc portion of human IgG1.

Additionally, trials of anti–TNF therapy in AS have yielded impressive results [21-25] and a recent systematic review and meta–analysis [26] described the benefits of anti–TNF therapy in patients with AS. However, only a small trial has reported on the efficacy of anti–TNF therapy for EAMs of AS [11], and further meta–analysis could strengthen this evidence. Therefore, we performed a meta–analysis of randomized clinical trials (RCTs) to provide an up–to–date and comprehensive picture of the clinical efficacy of anti–TNF therapy for the most common EAMs in patients with AS—uveitis, IBD and psoriasis.

## Methods

We captured all relevant studies published before February 2014 on MEDLINE, EMBASE and the Cochrane Library using following search terms: ‘ankylosing spondylitis’, ‘infliximab’, ‘etanercept’, ‘adalimumab’, ‘golimumab’, ‘certolizumab’, ‘TNF inhibitor/blocker/antagonists’ or ‘anti-TNF’.

Studies included in this meta-analysis met the following criteria: they were RCTs; their duration of study was ≥12 weeks; they used a parallel or crossover design of TNF inhibitor versus placebo treatment; and their data included information on uveitis, IBD and psoriasis in patients with AS.

Two independent investigators determined the relevance of the cited articles by reading the abstracts. Disagreements were resolved through discussion and consensus or, as needed, with consensus with a third investigator. When there were multiple articles from the same trial, the most complete and recently reported data were included.

Evaluations of methodological quality and risk of bias were performed independently by two reviewers, and disagreements between the two were resolved by consensus. Methodological quality of included articles was further assessed using modified Jadad criteria with an 8-item scale designed to assess randomization, blinding, withdrawals and dropouts, inclusion and exclusion criteria, adverse effects and statistical analysis [27]. Score range was from 0 (lowest quality) to 8 (highest quality). Scores of 4–8 denoted good to excellent (high quality) and 0–3 poor or low quality. Risk of bias was assessed according to the recommendations of the Cochrane Collaboration, classified as having either a low, high or unclear risk of bias [28,29]. Assessment of risk of bias in the included studies was based on sequence generation, allocation concealment, blinding (of participants, personnel and outcome assessors), incomplete outcome data, selective outcome reporting and other sources of bias, such as baseline imbalance and early stopping bias.

The statistical analysis was performed using Review Manager 5.2 (The Nordic Cochrane Centre, Copenhagen, Denmark) from the Cochrane Collaboration, 2013. Categorical dichotomous variables were assessed using the odds ratio (OR). *P* <0.05 was considered to be statistically significant; 95% confidence intervals (CI) were reported. Homogeneity was tested using the Q statistic (significance level at *P* < 0.10) and the *I*^2^ statistic (significance level at *I*^2^ > 50%) [30]. A random-effects model was used if the Q or *I*^2^ statistic was significant. Otherwise, a fixed-effects model was used. The existence of a publication bias for the meta-analysis was examined using a funnel plot. To assess the potential confounding effect of heterogeneity, subgroup analyses were performed. According to TNF-inhibitor classification, we divided those into two subgroups of TNF receptor fusion proteins (ETA) and monoclonal anti-TNF antibodies (INF, AND, GOL and CZP).

## Results

**Figure 1:**
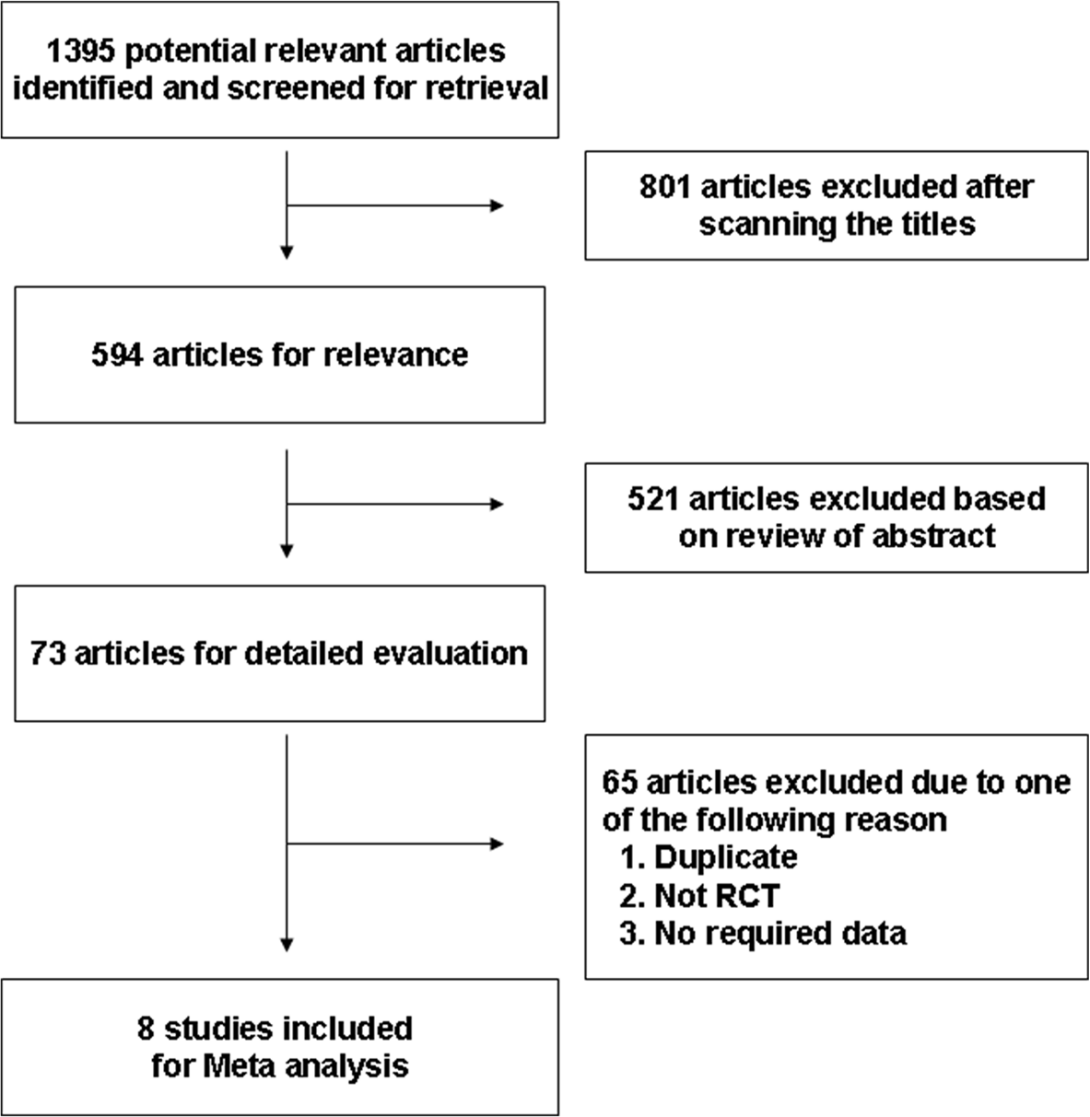
**Flow chart demonstrating the process of study selection.**

A total of 1,395 relevant articles were retrieved from various databases of which 801 were excluded after scanning the titles; 521 after carefully reading the abstracts; and an additional 65 articles for various reasons (duplicates, not RCT or no required data; Figure 1). Finally, 8 RCTs were retained for meta-analysis [31-38]. Overall, included studies were of adequate methodological quality (mean modified Jadad score 6.875 for included studies, and all 8 studies had a score ≥6). Included studies, basic characteristics of enrolled patients and details about drug therapy are presented in Table 1.

**Table 1:** **Basic characteristics of included studies**

**Study**	**No. of patients**	**Age (years)**	**Male patients N (%)**	**Duration of AS (years)**	**Study Duration (weeks)**	**Medications allowed during the study**	**Modified Jadad Score**	**Risk of bias**					
								**Sequence generation**	**Allocation concealment**	**blinding**	**Incomplete outcome data**	**Selective outcome reporting**	**other sources of bias**
ADA													
van der Heijde D. 2006 [31]	315				24	DMARDs, NSAIDs and glucocorticoids	6	?	?	√	√	√	√
ADA 40 mg every 2 weeks	208	41.7 ± 11.69	157 (75.5)	11.3 ± 9.99									
Placebo	107	43.4 ± 11.32	79 (73.8)	10.0 ± 8.34									
ETA													
Brandt J. 2003 [32]	30				24	NSAIDs	8	√	√	√	√	√	√
ETA 25 mg twice weekly	14	38.9 ± 9.1	10 (71.4)	14.9 ± 8.3									
Placebo	16	32.0 ± 7.5	12 (75)	11.4 ± 8.8									
Davis JC Jr. 2003 [33]	277				24	DMARDs, NSAIDs and glucocorticoids	6	?	?	√	√	√	√
ETA 25 mg twice weekly	138	42.1 (24–70)	105 (76)	10.1 (0–30.7)									
Placebo	139	41.9 (18–65)	105 (76)	10.5 (0–35.3)									
van der Heijde D. 2006 [34]	356				12	DMARDs, NSAIDs and glucocorticoids	6	?	?	√	√	√	√
ETA 25 mg twice weekly	150	39.8 ± 10.7	114 (76)	10.0 ± 9.1									
ETA 50 mg weekly	155	41.5 ± 11.0	109 (70)	9.0 ± 8.7									
Placebo	51	40.1 ± 10.9	40 (78)	8.5 ± 6.8									
IFX													
Braun J. 2002 [35]	69				12	NSAIDs	8	√	√	√	√	√	√
IFX 5 mg/Kg	34	40.6 ± 8.0	23 (68)	16.4 ± 8.3									
Placebo	35	39.0 ± 9.1	22 (63)	14.9 ± 9.3									
Marzo-Ortega H. 2005 [36]	42				30	NSAIDs, oral corticosteroids	8	√	√	√	√	√	√
IFX 5 mg/Kg + MTX	28	41 (28–74)	23 (82.14)	8 (0–41)									
Placebo + MTX	14	39 (30–56)	11 (78.57)	10 (0–35)									
GOL													
Inman RD. 2008 [37]	356				24	NSAIDs, MTX, SSA, HCQ, corticosteroids	7	?	?	√	√	√	√
GOL 50 mg	138	38 (29–46)	102 (73.8)	5.15 (1.60–11.60)									
GOL 100 mg	140	38 (30–47)	98 (70.0)	5.20 (1.50–13.25)									
Placebo	78	41 (31–50)	55 (70.5)	7.25 (2.80–18.60)									
CZP													
Landewé R. 2014 [38]	325				24	DMARDs, NSAIDs, MTX, SSA	6	?	?	√	√	√	√
CZP 200 mg every 2 weeks	111	39.1 ± 11.9	67 (60.4)	6.9 (0.3–34.2)									
CZP 400 mg every 4 weeks	107	39.8 ± 11.3	68 (63.6)	7.9 (0.3–44.8)									
Placebo	107	39.9 ± 12.4	65 (60.7)	7.7 (0.3–50.9)									

ADA = adalimumab; ETA = etanercept; IFX = infliximab; GOL = golimumab; CZP: certolizumab; MTX = methotrexate; DMARDs = disease-modifying antirheumatic drugs; NSAIDs = non-steroidal anti-inflammatory drugs; SSA = sulfasalazine; HCQ = hydroxychloroquine; √, low risk of bias; ?, unclear risk of bias.

The pooled analysis included 1,770 patients (1,223 randomized to anti-TNF therapy and 547 to placebo). Six trials [32-36,38] reported on uveitis that occurred in 7 patients in the anti-TNF therapy group and 16 in the placebo group; 5 trials [31,33,34,37,38] reported on IBD that occurred in 5 patients in the anti-TNF therapy group and 4 in the placebo group. No included trial reported on psoriasis.

**Figure 2:**
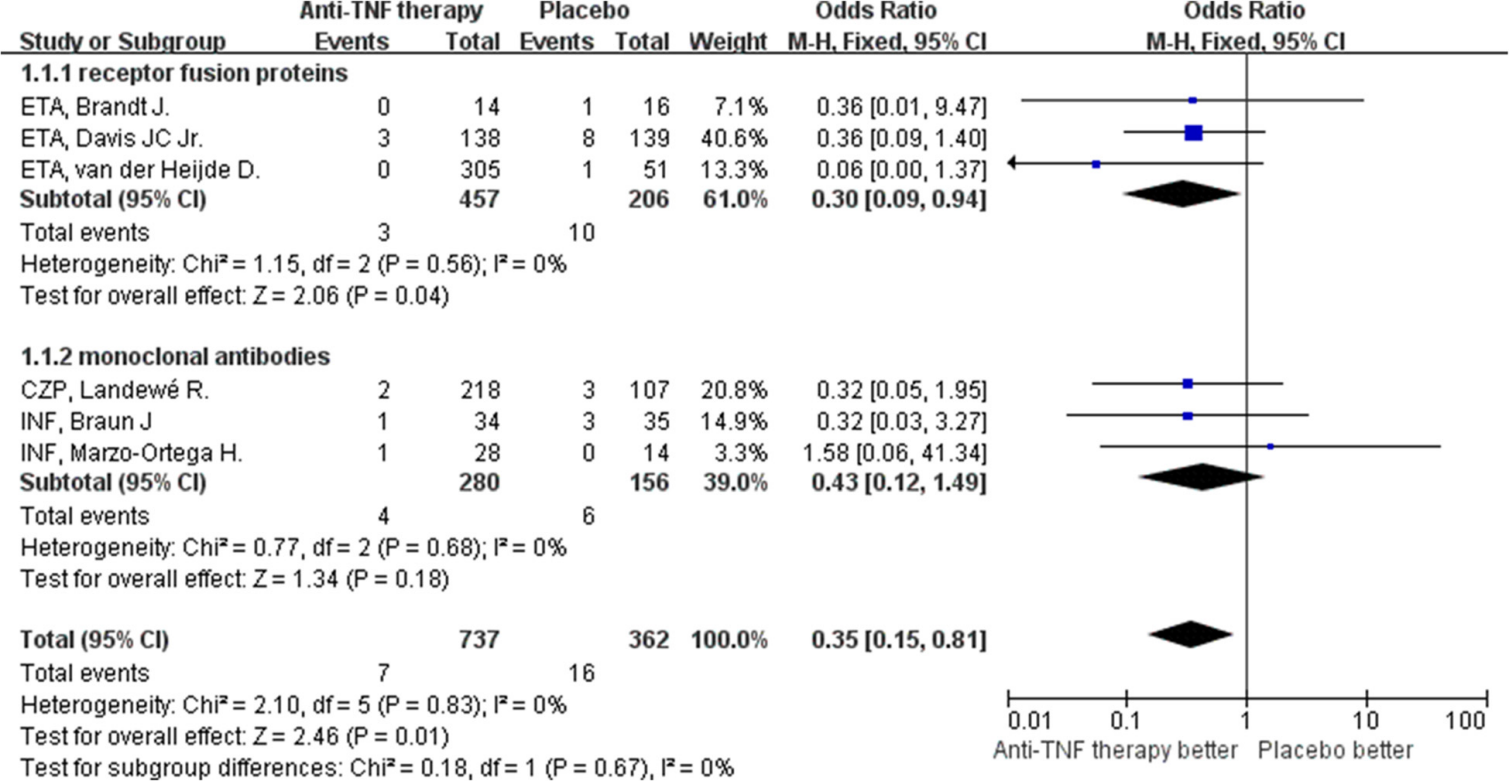
**Meta-analysis of uveitis between anti-TNF therapy and placebo for ankylosing spondylitis.**

**Figure 3:**
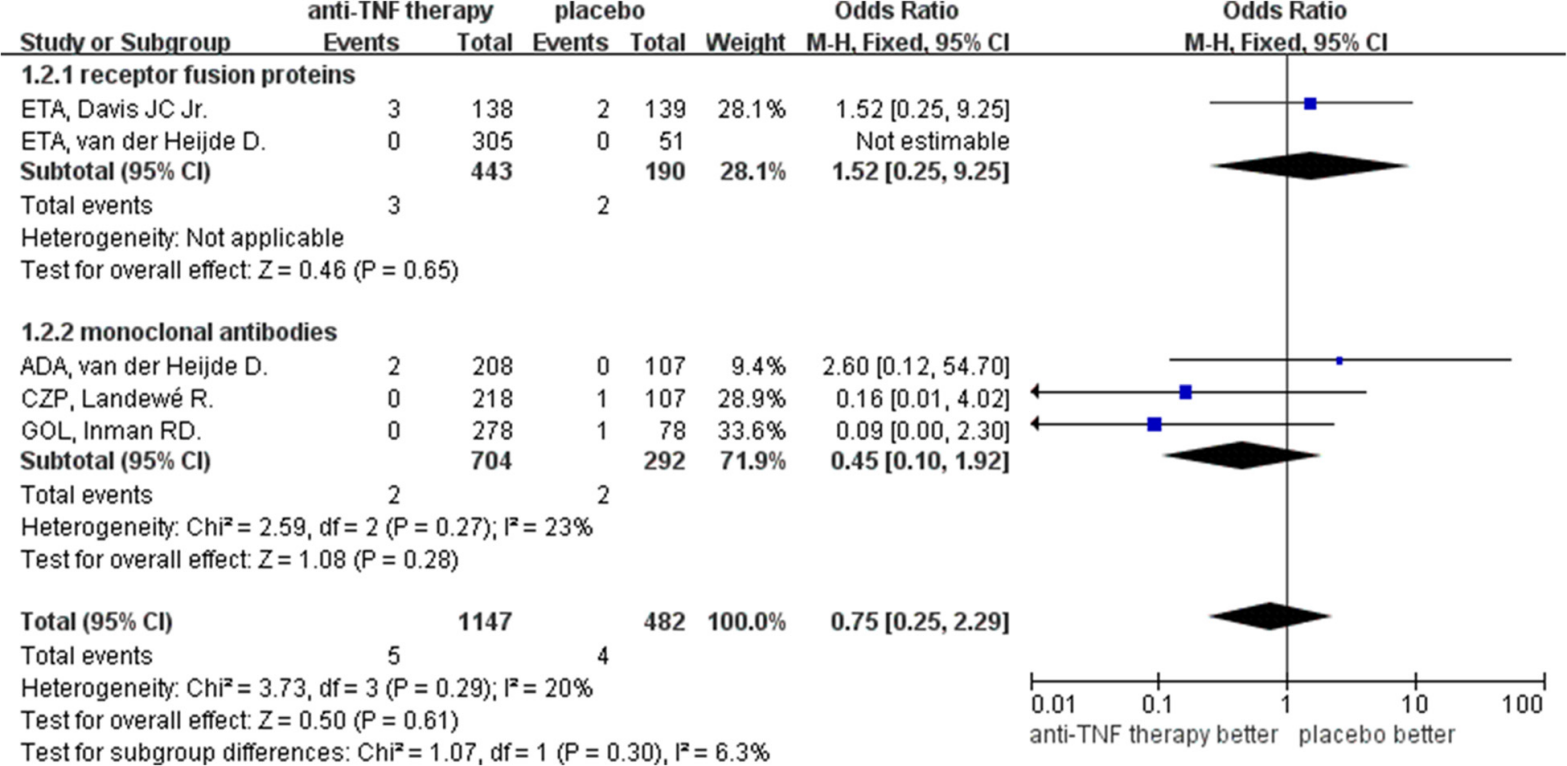
**Meta-analysis of inflammatory bowel disease between anti-TNF therapy and placebo for ankylosing spondylitis.**

**Figure 4:**
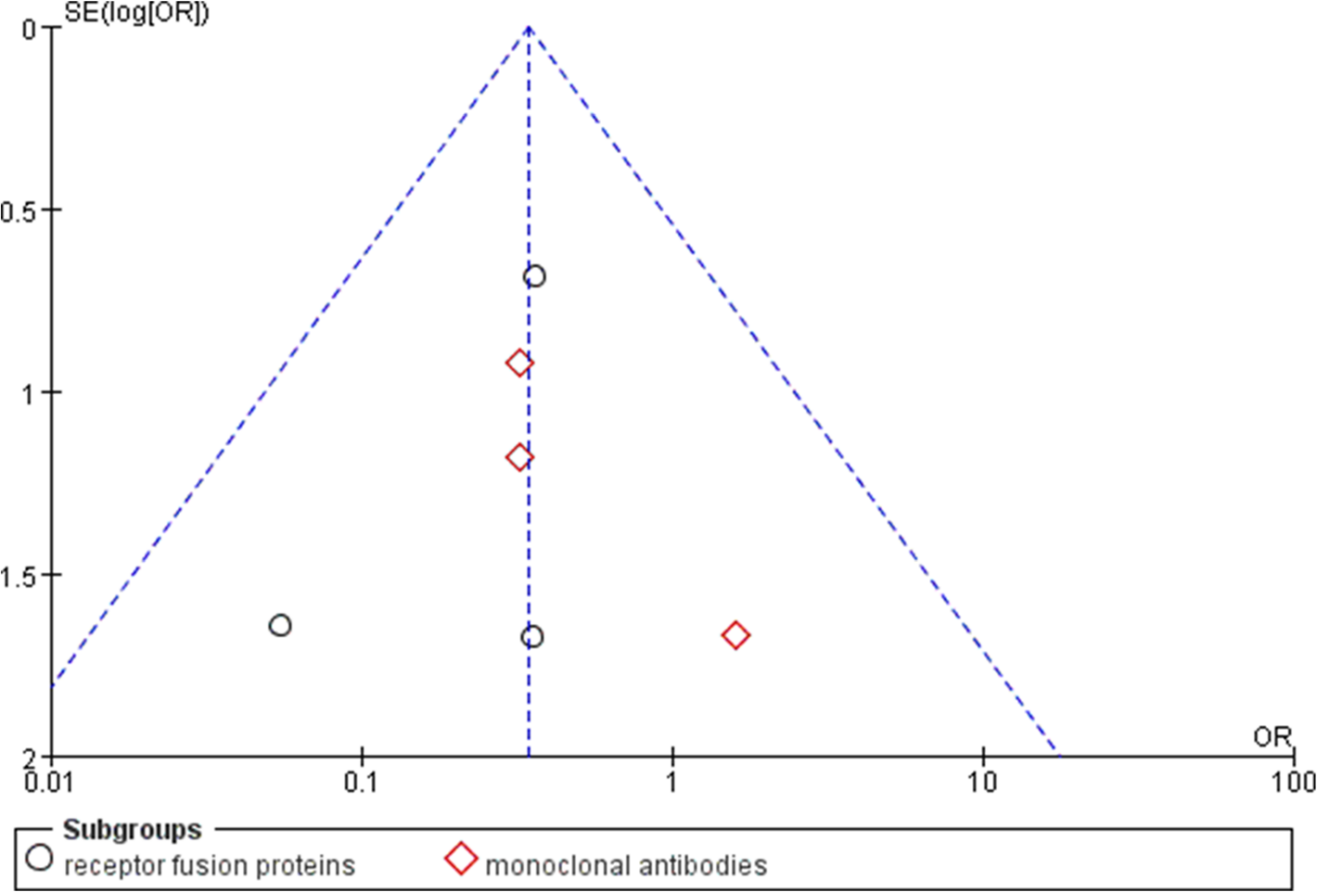
**Funnel plot of included trials that reported uveitis.**

**Figure 5:**
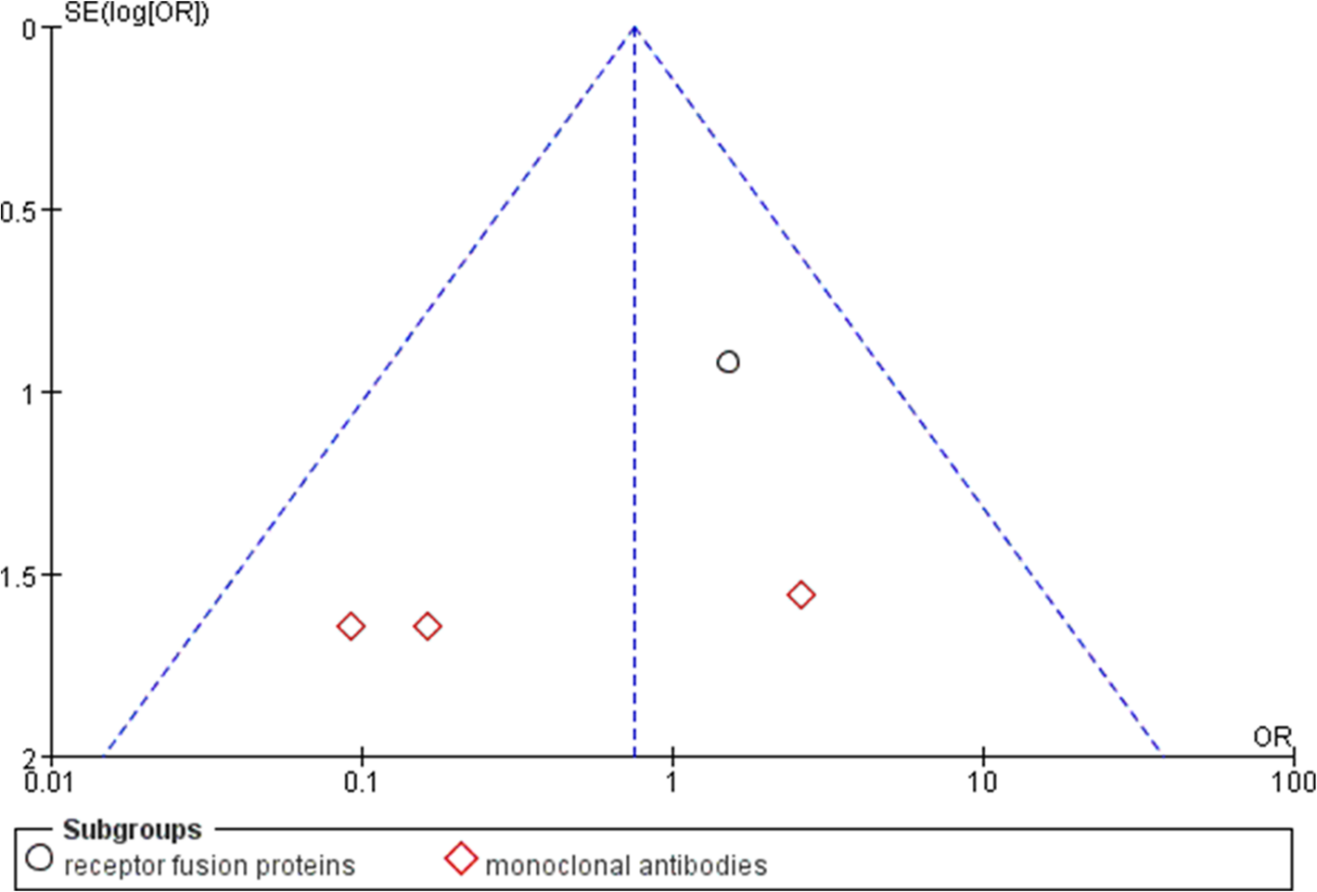
**Funnel plot of included trials that reported inflammatory bowel diseases.**

Anti-TNF therapy was associated with less uveitis than placebo in patients with AS (OR: 0.35, 95% CI: 0.15–0.81, *P* = 0.01, Figure 2). Subgroup analysis for uveitis in patients with AS showed TNF receptor fusion proteins to be more efficacious than placebo (OR: 0.30, 95% CI: 0.09–0.94, *P* = 0.04); whereas monoclonal anti-TNF antibodies did not significant differ from placebo (OR: 0.43, 95% CI: 0.12–1.49, *P* = 0.18). Analysis for IBD in these patients found that the anti-TNF therapy and placebo did not significantly differ (OR: 0.75, 95% CI: 0.25–0.29, *P* = 0.61, Figure 3); and neither monoclonal anti-TNF antibodies (OR: 0.45, 95% CI: 0.10–1.92, *P* = 0.28 versus placebo) or receptor fusion proteins (OR: 1.52, 95% CI: 0.25–9.25, *P* = 0.65 versus placebo) significantly differed from placebo. Funnel plot analysis showed symmetry, which indicates that publication bias was not a significant factor in these studies (Figures 4 and 5).

Analysis of risk of bias showed that only 3 trials reported their methods of sequence generation and allocation concealment in detail [32,35,36]. Blinding was performed properly in all included trials. All trials were free from incomplete outcome data and free from selective outcome reporting as well as other sources of bias. All 8 included trials had low or moderate risk of bias (Table 1).

## Discussion

This meta-analysis compared anti-TNF therapy with placebo in patients with AS. The results indicate significant positive benefits of anti-TNF agents to treat uveitis in these patients. For IBD treatment outcomes, the anti-TNF therapy group and the placebo group did not significantly differ. However, Subgroup analysis showed the receptor fusion protein ETA was more efficacious than placebo for uveitis in this patient population, whereas monoclonal anti-TNF antibodies were not. Neither monoclonal antibodies nor receptor fusion proteins significantly differed from placebo in treating IBD.

Anti-TNF therapy has been shown to be beneficial for the treatment of uveitis in patients with AS. A retrospective study [39] of patients with spondyloarthropathy further confirms the efficacy of anti-TNF therapy in reducing acute uveitis flares. Therefore, all available data imply that ETA would not be as effective as monoclonal anti-TNF antibodies [40-42]. However, our results differed. This discrepancy may reflect the biggest difference between our study and previous ones; we included trials that were all prospective RCTs whereas previous studies were almost all retrospective which tend to show larger risk values. Moreover, the mechanisms of anti-TNF antibodies and receptor fusion proteins are different; besides TNF-α, ETA also inhibits TNF-β. In an animal model of uveitis, higher TNF-β levels were found; ETA would therefore be expected to be even more effective [43]. More RCTs are required to further define the effect of ETA in AS patients with uveitis.

Braun *et al.* [44] investigated flare-ups or new-onset IBD in patients with AS who were treated with INF, ETA and ADA. New-onset and flares of IBD are infrequent in AS patients who receive anti-TNF therapy. The results showed that only INF and ADA might prevent IBD activity, both of which were associated with significant IBD rate reductions compared with ETA. The incidence of new-onset IBD in patients treated with placebo was not statistically different from that for any anti-TNF agent. ETA is not effective in controlling active CD [45]; in fact, cases have been reported of possible associated CD flare-ups [46] or new-onset CD [47] in AS patients undergoing ETA therapy. In our meta-analysis, we found that neither monoclonal anti-TNF antibodies nor TNF receptor fusion proteins were efficacious for IBD, but monoclonal anti-TNF antibodies had lower OR (implying greater efficacy) than TNF receptor fusion proteins. Only 5 small RCTs in our analysis had AS patients with IBD who were treated with anti-TNF agents. More RCT data is needed to establish the efficacy of anti-TNF antibodies for IBD in these patients.

Although anti-TNF agents are effective in treating skin and nail lesions of psoriasis [48,49], treatment with anti-TNF agents also can result in new manifestations of psoriasis for some patients [50]. We were unable to assess this in our meta-analysis because the included trails had no reported data of psoriasis.

The present study evaluated the efficacy of anti-TNF therapy on the frequency of EAMs in patients with AS. Anti-TNF therapy including ETA could be a credible alternative for AS patients who have uveitis. However, no anti-TNF therapy was efficacious for treating IBD in patients with AS. The 8 included studies that met the inclusion criteria had high-moderate Jadad scores; therefore the conclusions of this systematic analysis are reliable. More high-quality, large prospective RCTs with long-term follow-up are needed to confirm the efficacy and outcomes of anti-TNF therapy for EAMs of AS.

## Conclusions

Compared with placebo, anti-TNF therapy including ETA was associated with significantly fewer flares and new onset of uveitis, but were not significant efficacious for treating IBD in AS patients. This meta-analysis of patient-level data from 8 RCTs significantly advances the notion that anti-TNF therapy may be a credible alternative for AS patients with uveitis. Future studies involving anti-TNF therapy for EAMs of AS are needed.

## Abbreviations

TNF: Anti-tumor necrosis factor
AS: Ankylosing spondylitis
EAM: Extra-articular manifestation
RCT: Randomized controlled trial
IBD: Inflammatory bowel disease
CD: Crohn’s disease
UC: Ulcerative colitis
ASAS: Assessments in Ankylosing Spondylitis International Society
EULAR: European League against Rheumatism
NSAIDs: Non-steroidal anti-inflammatory drugs
DMARDs: Disease-modifying antirheumatic drugs
INF: Infliximab
IgG: Immunoglobulin G
ADA: Adalimumab
GOL: Golimumab
CZP: Certolizumab
ETA: Etanercept
OR: Odds ratio
CI: Confidence interval
HLA: Human leukocyte antigen
